# Treatment‐related mortality in newly diagnosed pediatric cancer: a population‐based analysis

**DOI:** 10.1002/cam4.1362

**Published:** 2018-02-23

**Authors:** Paul Gibson, Jason D. Pole, Tanya Lazor, Donna Johnston, Carol Portwine, Mariana Silva, Sarah Alexander, Lillian Sung, the International Pediatric Oncology Mortality Classification (IPOMC) Group

**Affiliations:** ^1^ Division of Haematology/Oncology Children's Hospital London Health Sciences Centre 800 Commissioners Rd E London Ontario N6A 5W9 Canada; ^2^ Pediatric Oncology Group of Ontario 480 University Ave Toronto Ontario M5G 1V2 Canada; ^3^ Program in Child Health Evaluative Sciences The Hospital for Sick Children Peter Gilgan Centre for Research and Learning 686 Bay St Toronto Ontario M5G 0A4 Canada; ^4^ Division of Hematology/Oncology Children's Hospital of Eastern Ontario 401 Smyth Rd Ottawa Ontario K1H 8L1 Canada; ^5^ Department of Pediatrics McMaster Children's Hospital 1200 Main St West Hamilton Ontario L8N 3Z5 Canada; ^6^ Department of Pediatrics Kingston General Hospital 76 Stuart St Kingston Ontario K7L 2V7 Canada; ^7^ Division of Haematology/Oncology The Hospital for Sick Children 555 University Ave Toronto Ontario M5G 1X8 Canada

**Keywords:** Canada, cancer, children, treatment‐related mortality

## Abstract

Using a previously developed reliable and valid treatment‐related mortality (TRM) definition, our objective was to describe the proportion of children newly diagnosed with cancer experiencing TRM and to identify risk factors for TRM in a population‐based cohort. We included children with cancer <19 years diagnosed and treated in Ontario who were diagnosed between 2003 and 2012. Children with cancer were identified using data in a provincial registry. Cumulative incidence of TRM was calculated where progressive disease death was considered a competing event. Among the 5179 children included, 179 had TRM, 478 died of progressive disease, and 4522 were still alive. At 5 years, the cumulative incidence of TRM among the entire cohort was 3.9% (95% confidence interval (CI) 3.3–4.5%). When compared to brain tumor patients, leukemia and lymphoma patients had a significantly higher risk of TRM (hazard ratio (HR) 2.5, 95% CI: 1.6–4.0; *P* < 0.0001). Infants were at significantly higher risk of TRM across diagnostic groups. Other factors associated with higher risks of TRM were metastatic disease (*P* < 0.0001), diagnosis prior to 1 January 2008 (*P* = 0.001), hematopoietic stem cell transplantation (HSCT) (*P* < 0.0001), and relapse (*P* < 0.0001). The 5‐year cumulative incidence of TRM was 3.9% among newly diagnosed children with cancer. Infants were at higher risk of TRM across diagnostic groups. Other risk factors for TRM were leukemia or lymphoma, metastatic disease, earlier diagnosis year, HSCT, and relapse. Future work should further refine prognostic factors by specific cancer diagnosis to best understand when and how to intervene to improve outcomes.

## Introduction

The prognosis for children with cancer diagnosed in Canada, the United States, and Europe is excellent, and most children will be cured 1. However, treatment‐related mortality (TRM) continues to be a major contributor to poor outcomes, particularly in those receiving intensive treatments 2, 3. We have previously argued that understanding the epidemiology of TRM is fundamental to improving outcomes 4.

In order to address the lack of consistency in TRM definitions, we developed a standardized TRM definition and cause‐of‐death attribution system 4. TRM is defined by the absence of progressive disease at the time of death. This approach was taken as many children with refractory disease will die from toxicities of therapy. The system is flexible as individual trials may choose to censor patients who relapse or those who undergo hematopoietic stem cell transplantation (HSCT). The developed TRM system was reliable and demonstrated criterion validity. We also found that trained clinical research associates (CRAs) could correctly apply the system with minimal training 4.

Using a population‐based data source in Ontario, we found that TRM was responsible for 26.4% of deaths in pediatric cancer 5. In this study, only patients who had died were included and they were divided into those who died from TRM versus progressive disease. Underlying diagnosis, younger age and absence of relapse were associated with TRM and causes of TRM differed by diagnosis group. However, this analysis was limited as it evaluated the proportion of deaths due to TRM, not the proportion of patients newly diagnosed with cancer who experience TRM. Thus, our objective was to describe the proportion of children newly diagnosed with cancer experiencing TRM and to identify risk factors for TRM in a population‐based cohort.

## Methods

Research Ethics Board approval was provided by The Hospital for Sick Children (SickKids), Toronto, Canada and the other four participating centers in Ontario. The requirement for informed consent was waived given the retrospective nature of the study.

### Setting and patients

The population consisted of children newly diagnosed with cancer and treated in the province of Ontario, Canada. There are five centers in Ontario that provide care for pediatric cancer patients, namely London Health Sciences Centre (London), Hamilton Health Sciences Centre (Hamilton), SickKids (Toronto), Cancer Centre of Southeastern Ontario at Kingston (Kingston), and Children's Hospital of Eastern Ontario (Ottawa). These centers report data to the Pediatric Oncology Group of Ontario Networked Information System (POGONIS), a population‐based provincial cancer registry. POGONIS captures 96–98% of children 0–14 years of age diagnosed with cancer when compared to the Ontario Cancer Registry 6. The capture rate for those 15–18 years is less complete because some adolescents are diagnosed and treated in adult facilities.

All patients diagnosed and reported to POGONIS between 1 January 2003 and 31 December 2012 were identified. Eligible children were <19 years of age at diagnosis, had an underlying diagnosis of cancer, and were diagnosed and treated at a POGO site. We excluded patients that had an unconfirmed cancer diagnosis. Children who died and who had not been seen within 1 year prior to death or those whose care was provided completely outside a POGO center were considered inevaluable and excluded from the analysis unless sufficient documentation existed to support a TRM designation.

### Treatment‐related mortality designation

With the classification system, deaths are classified as TRM or not TRM based upon the presence of progressive disease at the time of death 4. Deaths due to external causes such as accidents and suicides are considered to be TRM in this system. A flow diagram and educational material are available to facilitate training (https://www.sungresearch.com/trm-training-manual/). Causes of death are assigned only for TRM and not deaths due to progressive disease. For each TRM case, probable and possible causes of death are assigned based upon meeting specific criteria; a death may have multiple probable and possible causes of death. A primary cause of death is not assigned as it is often not possible to identify a single most likely cause of death.

### Procedure

Clinical research associates based at SickKids were trained in the TRM designation and cause‐of‐death attribution system 4. They traveled to each of the five Ontario institutions in order to classify all deaths as TRM or not TRM (progressive disease). The POGONIS database provided demographic data, cancer‐specific information, and outcomes for both participants who survived and who died.

### Statistics

We described the cumulative incidence of TRM among patients newly diagnosed with cancer and treated deaths from progressive disease as a competing event. Analyses were stratified by leukemia/lymphoma, solid tumor, and brain tumor; the risks of TRM were compared between groups using Gray's test. We evaluated the following factors to identify if they were associated with TRM: gender, age at diagnosis (as a continuous variable and <1, 1 to <5, 5 to <10, 10 to <15 and 15 to <19 years), metastatic disease at diagnosis, diagnosis before 1 January 2008 (midpoint of study), received surgery or radiotherapy, HSCT recipient (overall, allogeneic and autologous), enrolled on a therapeutic clinical trial at initial diagnosis, relapse, and census‐derived income quintile. Using the Statistics Canada Postal Code Conversion File software (PCCF+, Version 4J; Statistics Canada, Ottawa, Ontario, Canada), we linked the postal code at diagnosis to a 2001 census dissemination area. Dissemination areas are the smallest unit of geography defined by Statistics Canada; they include between 400 and 700 persons. From the 2001 census, income quintiles were determined that adjust for household size and regional differences 7. Effects were described using hazard ratios (HRs) with corresponding 95% confidence intervals (CIs). Statistical significance was defined as *P* value <0.05. Statistical analysis was conducted using the SAS statistical program (SAS‐PC, version 9.4; SAS Institute Inc, Cary, NC).

### Role of the funding source

The Pediatric Oncology Group of Ontario funded this study and had no role in study design, data collection, analysis, interpretation, writing of the report, or decision to submit the paper for publication.

## Results

**Figure 1:**
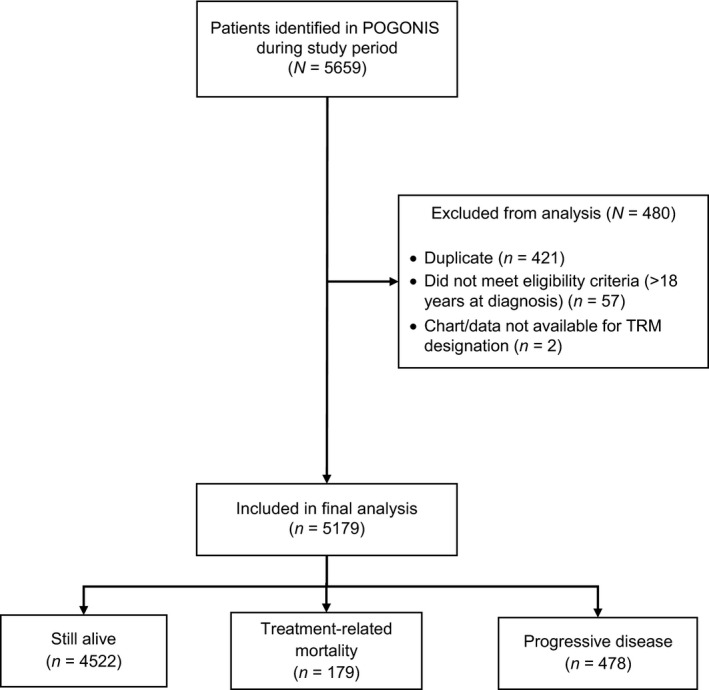
Flow diagram of participant identification, inclusion, and exclusion. POGONIS, Pediatric Oncology Group of Ontario Networked Information System.

There were 5179 children newly diagnosed with cancer during the study period. Figure 1 illustrates the breakdown of patients identified, reasons for exclusion from analysis, and final sample size based on status. A total of 179 children had a designation of TRM, 478 children died of progressive disease, and 4522 were still alive as of 31 December 2012.

Table 1 describes the demographics of the study population by full cohort and stratified by underlying primary diagnosis. The median age at diagnosis of the entire cohort was 7.0 years (range 0–18.99 years), and 952/5179 (18.4%) had metastatic disease at diagnosis. The cohort consisted of 2200 (42.5%) children with leukemia or lymphoma, 1919 (37.1%) with solid tumors, and 1060 (20.5%) with brain tumors. Table 2 summarizes the characteristics of children who were alive, died of progressive disease, and died of TRM when stratified by underlying diagnosis.

**Table 1:** Demographics of the study population

Characteristics	Total *N* = 5179	Leukemia or Lymphoma *N* = 2200	Solid Tumors *N* = 1919	Brain Tumors *N* = 1060
Demographics				
Male gender	2777 (53.6%)	1258 (57.2%)	938 (48.9%)	581 (54.8%)
Median age at diagnosis (Range)	7.0 (0.0, 18.99)	6.9 (0.0, 18.99)	6.0 (0.0, 18.8)	7.9 (0.0–18.8)
Age group				
< 1 year	493 (9.5%)	114 (5.2%)	336 (17.5%)	43 (4.1%)
1 to <5 years	1597 (30.8%)	746 (33.9%)	562 (29.3%)	289 (27.3%)
5 to <10 years	1070 (20.7%)	478 (21.7%)	269 (14.0%)	323 (30.5%)
10 to <15 years	1189 (23.0%)	485 (22.0%)	439 (22.9%)	265 (25.0%)
15 to <19 years	830 (16.0%)	377 (17.1%)	313 (16.3%)	140 (13.2%)
Metastatic disease at diagnosis	952 (18.4%)	420 (19.1%)	471 (24.5%)	61 (5.8%)
Diagnosis prior to 1 January 2008	2435 (47.0%)	1053 (47.9%)	902 (47.0%)	480 (45.3%)
Treatments				
Received surgery or radiotherapy	3081 (59.5%)	695 (31.6%)	1576 (82.1%)	810 (76.4%)
Received HSCT	495 (9.6%)	258 (11.7%)	150 (7.8%)	87 (8.2%)
Allogeneic	261 (5.0%)	226 (10.3%)	18 (0.9%)	17 (1.6%)
Autologous	234 (4.5%)	32 (1.5%)	132 (6.9%)	70 (6.6%)
Enrollment on clinical trial	1013 (19.6%)	676 (30.7%)	219 (11.4%)	118 (11.1%)
Outcomes				
Relapse	608 (11.7%)	253 (11.5%)	240 (12.5%)	115 (10.8%)
Median months diagnosis to death (IQR) (*n* = 657)	12.7 (5.5, 23.2)	10.3 (3.4, 20.7)	14.8 (7.2, 25.8)	11.8 (5.9, 19.8)
Socioeconomic				
Household income quintile				
1 (Lowest)	878 (17.0%)	380 (17.3%)	321 (16.7%)	177 (16.7%)
2	927 (17.9%)	405 (18.4%)	336 (17.5%)	186 (17.5%)
3	993 (19.2%)	413 (18.8%)	370 (19.3%)	210 (19.8%)
4	1172 (22.6%)	496 (22.5%)	434 (22.6%)	242 (22.8%)
5 (Highest)	1102 (21.3%)	477 (21.7%)	409 (21.3%)	216 (20.4%)
Missing	106 (2.1%)	29 (1.3%)	48 (2.5%)	29 (2.7%)

HSCT, hematopoietic stem cell transplant; IQR, interquartile range.

**Table 2:** Characteristics associated with treatment‐related mortality status stratified by disease type

Characteristics	Leukemia or Lymphoma *N* = 2200			Solid Tumors *N* = 1919			Brain Tumors *N* = 1060		
	Alive *N* = 1981	PD *N* = 99	TRM *N* = 120	Alive *N* = 1671	PD *N* = 212	TRM *N* = 36	Alive *N* = 870	PD *N* = 167	TRM *N* = 23
Demographics									
Male gender	1125 (56.8%)	61 (61.6%)	72 (60.0%)	799 (47.8%)	126 (59.4%)	13 (36.1%)	484 (55.6%)	87 (52.1%)	10 (43.5%)
Median age at diagnosis (Range)	6.9 (0.0–18.99)	7.4 (0.1–17.6)	7.0 (0.0–18.8)	5.9 (0.0–18.8)	7.8 (0.0–18.8)	2.2 (0.0–16.2)	8.4 (0.0–18.7)	6.9 (0.0–18.8)	3.4 (0.0–15.3)
Age group									
< 1 year	82 (4.1%)	14 (14.1%)	18 (15.0%)	297 (17.8%)	24 (11.3%)	15 (41.7%)	31 (3.6%)	7 (4.2%)	5 (21.7%)
1 to <5 years	696 (35.1%)	23 (23.2%)	27 (22.5%)	492 (29.4%)	61 (28.8%)	9 (25.0%)	237 (27.2%)	43 (25.7%)	9 (39.1%)
5 to <10 years	430 (21.7%)	21 (21.2%)	27 (22.5%)	230 (13.8%)	35 (16.5%)	4 (11.1%)	253 (29.1%)	64 (38.3%)	6 (26.1%)
10 to <15 years	437 (22.1%)	23 (23.2%)	25 (20.8%)	378 (22.6%)	54 (25.5%)	7 (19.4%)	229 (26.3%)	34 (20.4%)	2 (8.7%)
15 to <19 years	336 (17.0%)	18 (18.2%)	23 (19.2%)	274 (16.4%)	38 (17.9%)	1 (2.8%)	120 (13.8%)	19 (11.4%)	1 (4.3%)
Metastatic disease at diagnosis	365 (18.4%)	31 (31.3%)	24 (20.0%)	335 (20.0%)	119 (56.1%)	17 (47.2%)	39 (4.5%)	16 (9.6%)	6 (26.1%)
Diagnosis prior to 1 January 2008	914 (46.1%)	64 (64.6%)	75 (62.5%)	746 (44.6%)	131 (61.8%)	25 (69.4%)	356 (40.9%)	107 64.1%)	17 (73.9%)
Treatments									
Received surgery or radiotherapy	610 (30.8%)	51 (51.5%)	34 (28.3%)	1387 (83.0%)	168 (79.2%)	21 (58.3%)	645 (74.1%)	151 (90.4%)	14 (60.9%)
Received HSCT	163 (8.2%)	45 (45.5%)	50 (41.7%)	85 (5.1%)	58 (27.4%)	7 (19.4%)	70 (8.0%)	15 (9.0%)	2 (8.7%)
Allogeneic	139 (7.0%)	37 (37.4%)	50 (41.7%)	12 (0.7%)	5 (2.4%)	1 (2.8%)	17 (2.0%)	0 (0%)	0 (0%)
Autologous	24 (1.2%)	8 (8.1%)	0 (0%)	73 (4.4%)	53 (25.0%)	6 (16.7%)	53 (6.1%)	15 (9.0%)	2 (8.7%)
Enrollment on clinical trial	617 (31.1%)	26 (26.3%)	33 (27.5%)	171 (10.2%)	39 (18.4%)	9 (25.0%)	79 (9.1%)	35 (21.0%)	4 (17.4%)
Outcomes									
Relapse	135 (6.8%)	81 (81.8%)	37 (30.8%)	118 (7.1%)	118 (55.7%)	4 (11.1%)	52 (6.0%)	61 (36.5%)	2 (8.7%)
Median months diagnosis to death (IQR)	–	14.6 (8.8, 23.1)	7.1 (0.9, 18.4)	–	17.3 (10.5, 28.3)	1.1 (0.4, 8.6)	–	12.7 (7.4, 21.9)	0.5 (0.0, 6.8)
Socioeconomic									
Household income quintile									
1 (Lowest)	344 (17.4%)	15 (15.2%)	21 (17.5%)	265 (15.9%)	47 (22.2%)	9 (25.0%)	151 (17.4%)	21 (12.6%)	5 (21.7%)
2	359 (18.1%)	22 (22.2%)	24 (20.0%)	299 (17.9%)	29 (13.7%)	8 (22.2%)	146 (16.8%)	34 (20.4%)	6 (26.1%)
3	367 (18.5%)	16 (16.2%)	30 (25.0%)	312 (18.7%)	51 (24.1%)	7 (19.4%)	171 (19.7%)	34 (20.4%)	5 (21.7%)
4	448 (22.6%)	25 (25.3%)	23 (19.2%)	382 (22.9%)	49 (23.1%)	3 (8.3%)	196 (22.5%)	43 (25.8%)	3 (13.0%)
5 (Highest)	435 (22.0%)	21 (21.2%)	21 (17.5%)	367 (22.0%)	33 (15.6%)	9 (25.0%)	180 (20.7%)	33 (19.8%)	3 (13.0%)
Missing	28 (1.4%)	0 (0%)	1 (0.8%)	45 (2.7%)	3 (1.4%)	0 (0%)	26 (3.0%)	2 (1.2%)	1 (4.4%)

HSCT, hematopoietic stem cell transplant; IQR, interquartile range; PD,progressive disease; TRM, treatment‐related mortality.

**Figure 2:**
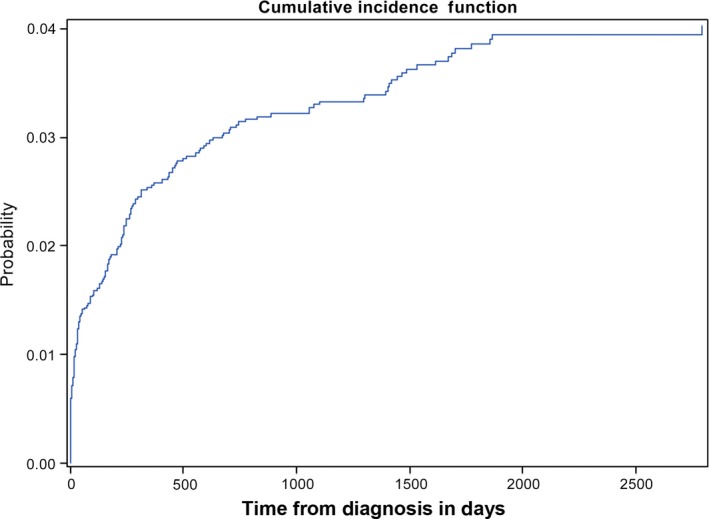
Cumulative incidence of treatment‐related mortality in the study cohort.

At 5 years, the cumulative incidence of TRM among the entire cohort was 3.9% (95% CI: 3.3–4.5%) (Fig. 2). When compared to brain tumor patients, leukemia and lymphoma patients had a significantly higher risk of TRM (HR = 2.5, 95% CI: 1.6–4.0; *P* < 0.0001) while solid tumor patients did not have a different risk of TRM (HR = 0.9, 95% CI: 0.5–1.4; *P* = 0.559). Cumulative incidence of TRM at 5 years for patients with leukemia and lymphoma was 6.2%, 95% CI: 5.3–7.4%, for solid tumors was 2.0%, 95% CI: 1.4–2.8%, and for brain tumors was 2.2%, 95% CI: 1.6–3.1%.

Table 3 describes factors associated with the cumulative incidence of TRM overall and stratified by underlying diagnosis in univariate analysis. Among the entire cohort, older age (as a continuous variable) was significantly associated with a lower risk of TRM (HR = 0.97, 95% CI 0.94, 0.996; *P* = 0.027) and the effect was significant in those with solid tumors and brain tumors. When divided by age category, all age categories had a significantly lower risk of TRM when compared to infants <1 year of age, among the entire group and when stratified by diagnosis type. When those 15 to <19 years of age were compared to those 5 to <10 years, TRM was not significantly different among the entire group (HR = 0.89, 95% CI: 0.54–1.47) or among leukemia or lymphoma (HR = 1.11, 95% CI: 0.64–1.92), solid tumor (HR = 0.22, 95% CI: 0.02–1.94), or brain tumor (HR = 0.39, 95% CI: 0.05–3.25) patients (data not shown). Other factors associated with higher risks of TRM were metastatic disease at diagnosis (*P* < 0.0001), diagnosis prior to 1 January 2008 (*P* = 0.001), absence of surgery or radiotherapy (*P* < 0.0001), HSCT overall (*P* < 0.0001), allogeneic HSCT (*P* < 0.0001), and relapse (*P* < 0.0001). There was no association between household income and TRM status.

**Table 3:** Factors associated with cumulative incidence of treatment‐related mortality

Characteristics	Total *N* = 5179 179 events		Leukemia or Lymphoma *N* = 2200 120 events		Solid Tumors *N* = 1919 36 events		Brain Tumors *N* = 1060 23 events	
	HR (95% CI)	*P* Value	HR (95% CI)	*P* Value	HR (95% CI)	*P* Value	HR (95% CI)	*P* Value
Demographics								
Male gender	0.98 (0.73, 1.32)	0.901	1.13 (0.79, 1.63)	0.504	0.59 (0.30, 1.17)	0.129	0.63 (0.28, 1.44)	0.275
Age at diagnosis	0.97 (0.94, 0.996)	0.027	0.99 (0.96, 1.03)	0.762	0.92 (0.86, 0.98)	0.014	0.82 (0.73, 0.93)	0.002
Age group								
< 1 year	REF		REF		REF		REF	
1 to <5 years	0.35 (0.23, 0.54)	<0.0001	0.20 (0.11, 0.37)	<0.0001	0.35 (0.15, 0.79)	0.012	0.26 (0.09, 0.77)	0.015
5 to <10 years	0.43 (0.27, 0.67)	0.0002	0.31 (0.17, 0.57)	0.0001	0.32 (0.11, 0.97)	0.044	0.15 (0.05, 0.49)	0.002
10 to <15 years	0.35 (0.22, 0.56)	<0.0001	0.29 ((0.16, 0.53)	<0.0001	0.35 (0.14, 0.85)	0.021	0.06 (0.01, 0.31)	0.0008
15 to <19 years	0.38 (0.23, 0.63)	0.0002	0.35 (0.19, 0.65)	0.0009	0.07 (0.01, 0.53)	0.010	0.06 (0.007, 0.50)	0.010
Metastatic disease at diagnosis	2.22 (1.56, 3.18)	<0.0001	1.43 (0.89, 2.29)	0.140	4.33 (2.02, 9.24)	0.0002	7.46 (2.82, 19.74)	<0.0001
Diagnosis prior to 1 January 2008	1.68 (1.23, 2.30)	0.001	1.33 (0.91, 1.95)	0.140	2.29 (1.14, 4.61)	0.020	2.97 (1.16, 7.63)	0.024
Treatments								
Surgery or radiotherapy	0.40 (0.30, 0.54)	<0.0001	0.79 (0.53, 1.16)	0.232	0.29 (0.15, 0.57)	0.0003	0.45 (0.20, 1.04)	0.061
Received HSCT	4.51 (3.33, 6.12)	<0.0001	5.16 (3.63, 7.35)	<0.0001	2.74 (1.23, 6.15)	0.014	1.01 (0.25, 4.48)	0.930
Allogeneic	7.39 (5.41, 10.09)	<0.0001	6.08 (4.28, 8.65)	<0.0001	2.84 (0.42, 19.25)	0.286	NA	
Autologous	0.95 (0.47, 1.92)	0.882	NA		2.62 (1.11, 6.20)	0.028	1.34 (0.32, 5.59)	0.692
Enrollment on clinical trial	1.40 (0.88, 2.24)	0.159	1.32 (0.77, 2.27)	0.310	1.35 (0.48, 3.78)	0.563	NA	
Outcomes								
Relapse	2.19 (1.57, 3.05)	<0.0001	3.16 (2.18, 4.58)	<0.0001	0.83 (0.30, 2.29)	0.714	0.73 (0.18, 3.03)	0.662
Socioeconomic								
Household income quintile								
1 (Lowest)	REF		REF		REF		REF	
2	1.04 (0.65, 1.64)	0.885	1.06 (0.59, 1.92)	0.836	0.86 (0.33, 2.22)	0.750	1.17 (0.36, 3.87)	0.789
3	1.06 (0.68, 1.66)	0.808	1.33 (0.76, 2.32)	0.320	0.67 (0.25, 1.80)	0.430	0.83 (0.24, 2.87)	0.772
4	0.62 (0.38. 1.01)	0.054	0.84 (0.46, 1.51)	0.550	0.25 (0.07, 0.91)	0.035	0.44 (0.10, 1.83)	0.257
5 (Highest)	0.74 (0.46, 1.19)	0.219	0.78 (0.42, 1.43)	0.416	0.79 (0.31, 1.99)	0.616	0.49 (0.12, 2.04)	0.326

HR, hazard ratio; CI, confidence interval; HSCT, hematopoietic stem cell transplant; NA, not available as model did not converge; REF, reference group.

Table 4 shows the ratio of deaths from TRM compared to progressive disease where 1 means the number of deaths due to treatment and cancer are equivalent and higher numbers indicate more deaths due to TRM. Among the entire cohort, the TRM to progressive disease death ratio was 0.37. In children with leukemia and lymphoma, the TRM ratio in infants was 1.29. In children with solid and brain tumors, ratios were generally low although the highest ratios were seen in infants with a ratio of 0.63 in solid tumors and a ratio of 0.71 in brain tumors.

**Table 4:** Treatment‐related mortality to progressive disease ratios

Characteristics	Leukemia or Lymphoma *N* = 2200		Solid Tumors *N* = 1919		Brain Tumors *N* = 1060		Overall *N* = 5179	
	Total	TRM Ratio	Total	TRM Ratio	Total	TRM Ratio	Total	TRM Ratio
Demographics								
Gender								
Male	1258	1.18	938	0.10	581	0.11	2777	0.35
Female	942	1.26	981	0.27	479	0.16	2402	0.41
Age group								
<1 year	114	1.29	336	0.63	43	0.71	493	0.84
1 to <5 years	746	1.17	562	0.15	289	0.21	1597	0.35
5 to <10 years	478	1.29	269	0.11	323	0.09	1070	0.31
10 to <15 years	485	1.09	439	0.13	265	0.06	1189	0.31
15 to <19 years	377	1.28	313	0.03	140	0.05	830	0.33
Metastatic disease at diagnosis								
Yes	420	0.77	471	0.14	61	0.38	952	0.28
No	1780	1.41	1448	0.20	999	0.11	4227	0.42
Diagnosis prior to January 1, 2008								
Yes	1053	1.17	902	0.19	480	0.16	2435	0.39
No	1147	1.29	1017	0.14	580	0.10	2744	0.35
Treatments								
Received surgery or radiotherapy								
Yes	695	0.67	1576	0.13	810	0.09	3081	0.19
No	1505	1.79	343	0.34	250	0.56	2098	1.02
Received HSCT	258	1.11	150	0.12	87	0.13	495	0.50
Allogeneic	226	1.35	18	0.20	17	NA	261	1.21
Autologous	32	0.00	132	0.11	70	0.13	234	0.11
Enrollment on clinical trial								
Yes	676	1.27	219	0.23	118	0.11	1013	0.46
No	1524	1.19	1700	0.16	942	0.14	4166	0.35
Outcomes								
Relapse								
Yes	253	0.46	240	0.03	115	0.03	608	0.17
No	1947	4.61	1679	0.34	945	0.20	4571	0.62
Socioeconomic								
Household income quintile								
1 (Lowest)	380	1.40	321	0.19	177	0.24	878	0.42
2	405	1.09	336	0.28	186	0.18	927	0.45
3	413	1.88	370	0.14	210	0.15	993	0.42
4	496	0.92	434	0.06	242	0.07	1172	0.25
5 (Highest)	477	1.00	409	0.27	216	0.09	1102	0.38

HSCT, hematopoietic stem cell transplant; IQR, interquartile range; PD, progressive disease; TRM, treatment‐related mortality; NA, not applicable.

## Discussion

In this population‐based analysis of newly diagnosed children with cancer, we found that overall, the cumulative incidence of TRM at 5 years was 3.9% and was highest among children with leukemia and lymphoma. Risk factors for TRM were younger age, metastatic disease, earlier diagnosis year, and HSCT. Household income was not associated with TRM. Given that children with cancer have an overall survival in excess of 82% 8, 9, this study suggests that elimination of TRM would meaningfully improve outcomes for the 18% of children who do not survive.

We also presented a novel approach to displaying TRM rates, namely the TRM to progressive disease death ratio. The rationale behind this ratio is that description of TRM alone does not place the TRM rate in context. For example, in poor prognosis cancers, increased TRM may be acceptable if it achieves more cures. However, if more children are dying from TRM compared with progressive disease, supportive care should be enhanced and treatment intensity reduction could be considered. This data may also help to justify additional resources such as hospitalization during neutropenia. On the other hand, if the progressive disease death rate is higher than TRM, then this suggests that intensification of therapy may be appropriate for cancers which are sensitive to chemotherapy. If the rates are similar but overall survival is poor, this may suggest that innovative approaches are required. However, as with any new metric, interpretation of the ratio will require further application and input from stakeholders. Nonetheless, a ratio exceeding 1 means more deaths are due to TRM than progressive disease and may be an important benchmark.

We found that patients with leukemia or lymphoma were at the highest risk of TRM. This finding is not surprising given that several studies have identified children with acute lymphoblastic leukemia 2, 10, acute myeloid leukemia 11, 12, 13, and some non‐Hodgkin's lymphomas as being at higher risk of toxic mortality due to receipt of intensive highly myelosuppressive chemotherapy in addition to allogeneic HSCT 14. The associations between allogeneic HSCT, metastatic disease, and relapse with more TRM are also not surprising and likely reflect administration of intensive therapy. We also found that receipt of surgery or radiotherapy was associated with a lower risk of TRM. This finding is likely confounded by underlying diagnosis since patients with solid tumors and brain tumors had lower TRM rates and they are the patients more likely to undergo surgical procedures for the removal of cancer and radiotherapy.

Although we found that older age was associated with a lower risk of TRM, the higher risk was focused on the infant population and not distributed across age groups. This finding is in contrast to several reports. Increased TRM was observed in adolescents 15–18 years of age with acute lymphoblastic leukemia when compared to younger children 15. Similarly, in a study of patients with acute myeloid leukemia, adolescents and young adults (16 years of age and older) were found to be at significantly higher risk of TRM 16. However, these reports included few or no infants and our study does not include patients older than 18 years of age. Both of these factors limit the comparability of other studies to ours.

A strength of this study is the use of consistent and trained CRAs to abstract the TRM data. A second strength is its population‐based nature which improves generalizability and avoids selection bias as patients with comorbidities and those who present critically ill are often excluded from trials. However, our findings have several limitations. We lack detailed potential prognostic factors which could be important such as facility‐ and provider‐related data. Second, our cohort only extends to <19 years of age and thus, we do not have TRM data for young adults, a subgroup of patients of particular importance given known disparities based on survival and enrollment on clinical trials 17. Third, we did not include cause of death in this manuscript. We plan to explore cause of death within specific patient subgroups such that disease‐specific characteristics can be evaluated as potential prognostic factors.

In conclusion, the cumulative incidence of TRM was 3.9% among newly diagnosed children with cancer. Infants were at higher risk of TRM across diagnostic groups. Other risk factors for TRM were leukemia or lymphoma, metastatic disease, earlier diagnosis year, HSCT, and relapse. Future work should further refine prognostic factors by specific cancer diagnosis to best understand when and how to intervene to improve outcomes.

## Conflict of Interest

No authors have a conflict of interest to declare.
